# Localization of early infarction on non-contrast CT images in acute ischemic stroke with deep learning approach

**DOI:** 10.1038/s41598-023-45573-7

**Published:** 2023-11-09

**Authors:** Sulagna Mohapatra, Tsong-Hai Lee, Prasan Kumar Sahoo, Ching-Yi Wu

**Affiliations:** 1 Department of Computer Science and Information Engineering, Chang Gung University, 259, Wen-Hwa 1st Road, Guishan, Taoyuan, 33302 Taiwan; 2 Department of Neurology, Chang Gung Memorial Hospital, Linkou Medical Center, No. 5, Fu-Hsing street, Guishan, Taoyuan, 333 Taiwan; 3 College of Medicine, Chang Gung University, Taoyuan, Taiwan; 4 Department of Occupational Therapy and Graduate Institute of Behavioral Sciences, College of Medicine, Chang Gung University, Taoyuan, Taiwan

**Keywords:** Neuroscience, Medical research, Neurology, Engineering, Mathematics and computing

## Abstract

Localization of early infarction on first-line Non-contrast computed tomogram (NCCT) guides prompt treatment to improve stroke outcome. Our previous study has shown a good performance in the identification of ischemic injury on NCCT. In the present study, we developed a deep learning (DL) localization model to help localize the early infarction sign on NCCT. This retrospective study included consecutive 517 ischemic stroke (IS) patients who received NCCT within 12 h after stroke onset. A total of 21,436 infarction patches and 20,391 non-infarction patches were extracted from the slice pool of 1,634 NCCT according to brain symmetricity property. The generated patches were fed into different pretrained convolutional neural network (CNN) models such as Visual Geometry Group 16 (VGG16), GoogleNet, Residual Networks 50 (ResNet50), Inception-ResNet-v2 (IR-v2), Inception-v3 and Inception-v4. The selected VGG16 model could detect the early infarction in both supratentorial and infratentorial regions to achieve an average area under curve (AUC) 0.73 after extensive customization. The properly tuned-VGG16 model could identify the early infarction in the cortical, subcortical and cortical plus subcortical areas of supratentorial region with the mean AUC > 0.70. Further, the model could attain 95.6% of accuracy on recognizing infarction lesion in 494 out of 517 IS patients.

## Introduction

Stroke is the second leading cause of death and most significant disability in the world^1^. Cerebral infarction occupies approximately 80% of total strokes and is due to insufficient blood supply to the brain, leading to the death of brain tissue. In acute ischemic stroke (IS), the treatment with intravenous recombinant tissue plasminogen activator within 3–4.5 h and intra-arterial mechanical thrombectomy within 6–24 h has been well advised in stroke guideline^2^. Early identification of ischemic size and location on brain images can help decision-making on urgent treatment of acute ischemic stroke. NCCT is the most commonly used brain image due to its well accessibility with versatile fast speed. However, NCCT has the limitation in early IS (EIS) lesion localization, which may take hours to days to be visible on NCCT depending on the stroke duration, severity and location^3,4^, especially in the infratentorial region such as medulla, pons, midbrain, and cerebellum (Supplementary Fig. S1). MRI can give a better localization of infarction at early hours after stroke onset, but MRI is expensive, time-consuming and not readily available in most hospitals^5,6^.

Since the treatment time window for acute IS is narrow, urgent detection and localization of early IS on NCCT are highly demanded to save time and improve treatment outcome. Artificial intelligence has been widely used in medical image data analysis^7–10^. With the potential of machine learning (ML), automated software named as e-ASPECTS (Alberta Stroke Program Early Computed Tomography Score) and RAPID ASPECTS (iSchemaView) have been developed to analyze the NCCT and quantify the ASPECT score automatically in early IS^11–13^. In the case of ML, when big data is involved, it becomes a cumbersome job to extract the features manually even when an expert is involved. Besides, ASPECTS focuses mainly on the ten regions of middle cerebral artery (MCA) area in the supratentorial region without considering the areas of anterior cerebral artery (ACA) and posterior cerebral artery (PCA)^14^ (Supplementary Fig. S2). Further, ASPECTS scoring needs experience and has limited applicability in detecting small infarction such as lacunar size ≤ 1.5 cm. In addition, the localization of infarction using NCCT in cortical area is challenging in comparison to subcortical area due to the presence of central fissure and sulci (Supplementary Fig. S3).

Although few related works developed the early ischemic stroke detection and segmentation models using the first-line NCCT images, none of them considered the analysis based on different region of occurrence as the complicacy of detection varies with the area and size of the infarction^15–23^. For instance, early ischemic lesion detection for stroke onset < 9 h was performed for a small study population of 116 patients^15^. Although, the model achieved an accuracy of 0.74, the detection was limited to anterior and posterior territories only. A context-aware CNN network proposed for early ischemic stroke sign detection^16^ (< 6 h of stroke onset) to estimate the presence of ischemic stroke sign at the hemisphere level from 170 patients data. However, it was not a robust method as the ischemic stroke could occur at any part of the brain. Further, a DL-based early infarct identification and ASPECT scoring determination using NCCT for 260 numbers of ischemic patients was proposed^17^. The designed model considered only the MCA region and achieved an accuracy = 0.85 and AUC = 0.83. However, the lower F-score = 0.40 signifies the imbalance outcome of precision and recall. An early stroke detection method using YOLO v3 was developed for 238 patients collected from two institutions^18^. Although, the model included the cases of smaller size of infarction, the value of F-score < 0.50 was due to low sensitivity (0.40) and precision (0.60). The CNN framework designed for ischemic stroke detection achieved 90% accuracy^19^ by considering very less number of data set (256 patches). Besides, the collected data were from the MCA territory of the supratentorial region only and did not focus on the stroke localization.

Apart from the CNN analysis, several methods developed the ischemic region localization using the concept of ML and statistical analysis^20,21^. One of them developed the early infarction (< 6 h of onset) detection method from the NCCT by considering the infarction occurred on the M1 segment of MCA^20^. Even if the considered stroke age was < 6 h, the infarction region on NCCT was visible. Although, the ML-based automatic ASPECT prediction model^21^ achieved an accuracy greater than 0.80, the sensitivity was only 0.50 for different parts of MCA regions such as M1, M3, M4, M6, caudate and internal capsule. The mathematical models^22,23^ developed for ischemic region detection and localization by calculating the stroke imaging marker (SIM) manually. However, the manual calculation of early IS based on single parametric value could not be considered as a general solution for the extensive amount of data. Besides, the intensive mathematical calculation requires massive computational time and needs the modeler to understand the relation between parameters before using it for further analysis.

Some researchers developed AI-based automatic segmentation of ischemic region by considering the MR images. For early detection of ischemic stroke, authors proposed a fully automatic CNN system by considering Diffusion Weighted Imaging (DWI)^5^. The proposed CNN model achieved an average dice score 0.67 with generation of higher False Negatives (FNs). This could lead to misclassification when the brain contains the only lesion. A residual-structured fully convolutional network (Res-FCN) was developed for automatic segmentation of acute and sub-acute ischemic stroke by considering different MRI sequences such as DWI, ADC (Apparent Diffusion Coefficient) and T2^24^. However, the designed model has very low training and testing accuracy of 0.80 and 0.64, respectively. One study achieved sensitivity = 0.93 and specificity = 0.82 from the designed 3D CNN model by considering the CT angiography (CTA) images for the acute ischemic stroke detection^25^. Nonetheless, the use of injected material for CTA images may bring lots of side effects such as itching, vomiting, nausea and also the chances of cancer. Therefore, for faster and safe ischemic stroke diagnosis, we considered the affordable first-line NCCT for our analysis.

Our previous study has shown the customized-VGG16 CNN model can perform well to identify the presence of early ischemic lesions on NCCT slices using the concept of automatic feature learning^3^. The present study intended to develop an automatic localization model for early infarction sign irrespective of any cerebral region on NCCT examined within 12 h after stroke onset.

## Methods

### Study population

**Figure 1:**
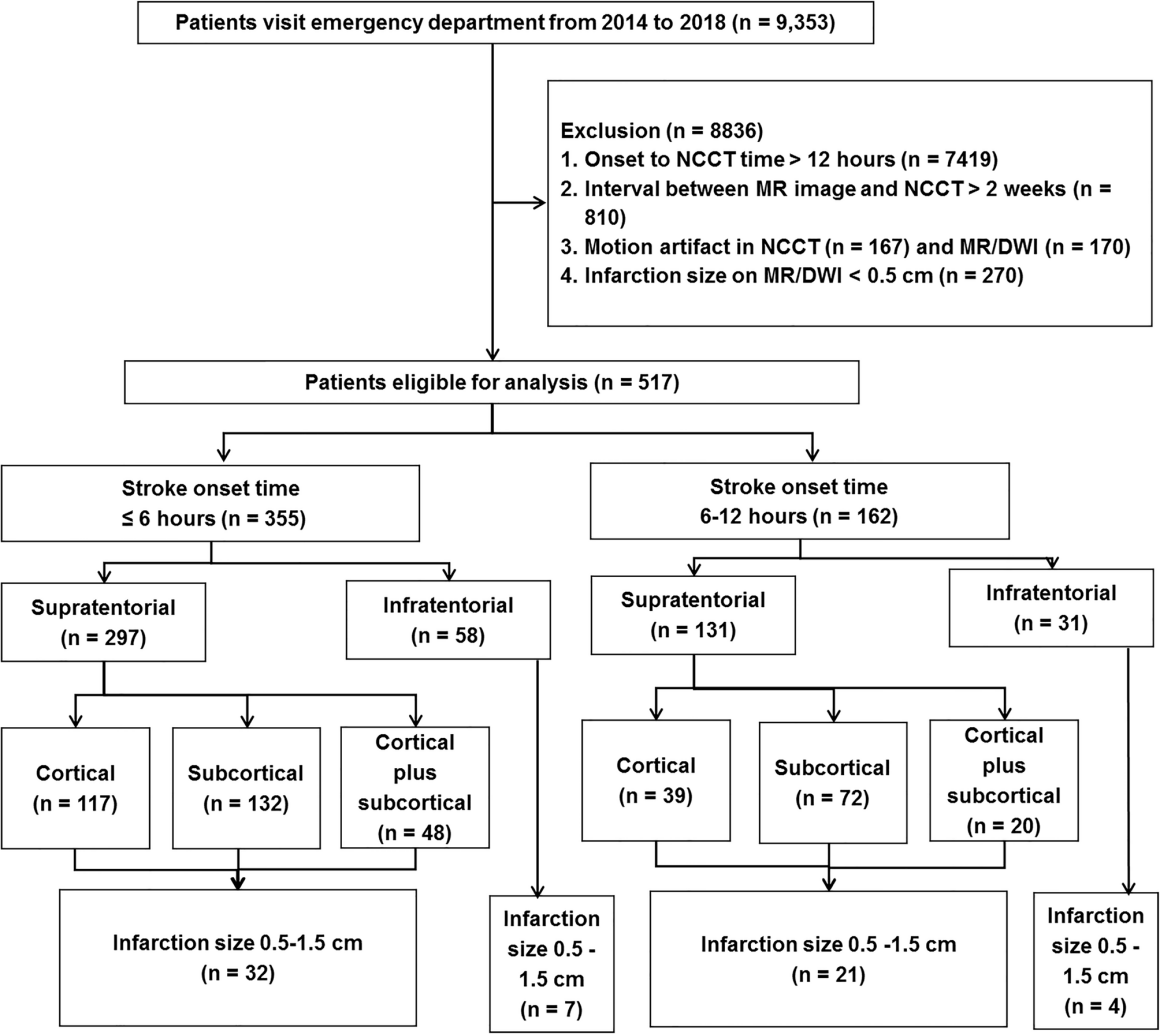
Patient recruitment flowchart. The figure represents the inclusion and exclusion criteria of the ischemic stroke patients enrolled and considered for the present analysis according to their stroke onset time, affected brain regions, areas and size of infarction. *NCCT* non-contrast computed tomogram, *MR* magnetic resonance, *DWI* diffusion-weighted image.

A total of 9,353 IS patients were retrospectively screened from 2014 to 2018 at Chang Gung Memorial Hospital, Linkou Medical Center, Taiwan. Among them, 517 IS patients (5.52%) met the inclusion criteria and were recruited for further processing (Fig. 1). Both NCCT and MRI were collected after de-identification with the imaging interval < 14 days (mean ± SD = 7.4 ± 5.3 days), and there was no recurrent ischemic event during this interval. The MR/DWI sequences were used for image annotation, while MR/ADC sequences were employed to validate the ischemic region in DWI. The images were collected from Chang Gung Research Databank in the format of Digital Imaging and Communications in Medicine (DICOM) with each image size 512 × 512 pixels. The study was approved by the Institutional Review Board (IRB) of the Chang Gung Medical Foundation, Taipei, Taiwan with license number 201900028B0. The informed consent was waived by the Chang Gung Medical Foundation, Institutional Review Board, 199, Tung Hwa North Road, Taipei, Taiwan, 10507, Republic of China. All methods were performed in accordance with the relevant guidelines and regulations.

Brain CT scans were performed on a single detector CT scanner (Aquilion 64, Toshiba, Japan). The thickness of each brain NCCT was 5 mm. The HU of original NCCT was transformed from a brain/sinus window (center 40HU, width 150HU) into 256 Gy levels. Brain MR image was performed at a 3.0 Tesla scanner (Ingenia 3.0T MR system, Philips, USA). The eligible images were screened based on the regular reports by neuroradiologists who identified no infarction on initial NCCT which was examined within 12 h after stroke onset but positive DWI/ADC signal on subsequent MRI which was re-confirmed by two neurologists. In case of conflict between neuroradiologists and neurologists, the images were not included for analysis (the inter-observer difference near 100%).

### Study methodology

Five phases were performed to establish the infarction localization model including preprocessing, ground truth formation, CNN input preparation, infarction sign detection, and infarction localization (Supplementary Fig. S4).

### Preprocessing phase

To improve the issues of low resolution, poor contrast quality, presence of skull bone, and in-built noise that could create the difficulty in detecting the infarction region, the following preprocessing steps were used. First, the NCCT DICOM images were converted to JPEG (joint photographic expert group) using the software RadiAnt DICOM Viewer^26^ with the maintenance of the original image dimension 512 × 512 and the standard 8-bit grayscale depth (0–255). A pixel-level analysis was performed instead of voxel-level for which 2D NCCT slices were preferred^27^. The distortion of brain tissue was carefully prevented after the conversion of NCCT images.

Second, the NCCT slices containing infarction were differentiated from those with no infarction based on DWI/ADC sequence. The mapping between NCCT and MRI was performed considering various cerebral features including the structure of ventricle, sulcus and order of the image sequences. Third, bony skull and falx calcification were removed by combining the automatic algorithms such as binary and pixel-based thresholding along with the combination of morphological operations like erosion and opening both together (https://www.mathworks.com/help/images/morphological-dilation-and-erosion.html). Fourth, to increase the contrast quality as well as to remove the inbuilt noise from NCCT, the Denoising Convolutional Neural Network (DnCNN) (https://www.mathworks.com/help/images/ref/dncnnlayers.html) was applied in the final step of the preprocessing after comparing the Peak-Signal-to-Noise Ratio (PSNR) value with different filtering algorithms such as mean filter, median filter, etc. (https://www.mathworks.com/help/images/noise-removal.html).

### Ground truth formation phase

To prevent the manual labelling errors, the DWI/ADC sequence was used as a reference to create a label on NCCT by using supervised learning method^28^. However, several intermediate processing steps such as brain tissue tilt adjustment, cropping and resizing were performed using ImageJ software^29^ prior to the annotation. These processing steps were necessary as the acquisition settings and the patient health condition vary with both modalities. However, these intermediate processing were solely performed for the annotation of the training images. First, the tilt adjustment was done on the selected NCCT and DWI slices to make them completely straight by rotating clockwise or anti-clockwise until the cerebral falx line of both the image modalities form 90° or 270° angle with the x-axis and a 0° or 180° angle with the y-axis. This angular adjustment was performed automatically using bilinear interpolation method embedded in ImageJ. In the next step, the brain tissue part was cropped from both images. Further, the cropped NCCT slices were resized equal to the size of DWI to match the accurate region of infarction. Then, the infarction region was extracted from the DWI/ADC image using the Shanbhag segmentation method embedded inside the ImageJ. Next, the masked infarction region was overlaid on the corresponding preprocessed NCCT. Finally, the NCCT with annotated early infarction was confirmed by neurologists using corresponding DWI/ADC. The T2 shine-through effect of DWI slice was taken care of by the corresponding ADC slice.

### CNN input preparation phase

The DL-based infarction localization model considered the image patches as the input to the CNN instead of the entire NCCT slices. The use of image patches was to prevent from the imbalanced pixel ratios between the acute infarction lesion and the normal brain region. To prepare the appropriate input for the CNN model, different sub-phases such as patch generation, patch selection and patch resizing were adopted in this phase.

For patch generation, TileMage Image Splitter version 2.11 (https://tilemage-image-splitter.en.uptodown.com/windows) was used to divide the image slices into smaller patches of the user-defined size, where the size of patches varied (15–22 pixels) based on the dimension of the input image. The patches were formed considering both the annotated and its corresponding un-annotated NCCT. The generated patches were stored in JPEG format based on the requirement of the DL-based localization model (Supplementary Fig. S5a).

For patch selection, both infarction and non-infarction patches were selected for AI analysis. In the designed model, the infarction (abnormal) patches were extracted from the infarction region whereas the non-infarction (normal) patches were collected from the brain region situated at the contralateral hemisphere by applying the brain symmetry property (Supplementary Fig. S5b). For those patients who had infarction on both hemispheres, the non-infarction patches from both hemispheres were considered for training.

For patch resizing, the pools of infarction and non-infarction patches were resized before testing in the DL models. The resizing for a batch of patches was performed using the Plastiliq Image Resizer version 1.2.5 (https://plastiliq-image-resizer.en.uptodown.com/windows) (Supplementary Fig. S5c).

### Infarction sign detection phase

The infarction localization phase focused mainly on the identification of infarction region that obtained using CNN model selection and finalization. The infarction identification process was carried out by correctly classifying the infarction and non-infarction patches using pretrained CNN. For this purpose, a total of 21,436 infarction (abnormal) patches and 20,391 non-infarction (normal) patches were extracted from the 1,634 NCCT slices of 517 patients. The main aim of this localization phase was to identify at least a single infarction patch accurately that could assist the diagnosis of acute cerebral infarction.

For CNN model selection and input patch size, the entire pool of both abnormal and normal patches was divided randomly into training/validation and testing sets in the ratio of 80:20. Several state-of-the-art pretrained CNN models that were already trained with a large ImageNet dataset^30^ were employed based on their reusability and faster analysis. The pretrained CNN models adopted the concept of transfer learning^31^, where the learning process of those pretrained models was initiated from the patterns which were already learned during the training of various dataset instead of learning from scratch. Different pretrained CNN models were performed including Visual Geometry Group (VGG16)^32^, Residual Networks 50 (ResNet50)^33^, GoogleNet^34^, Inception-v3^35^, Inception-v4^36^, and Inception-ResNet-v2 (IR-v2)^36^ that were trained on ImageNet dataset and were customized using transfer learning.

For CNN model finalization, after selection of the appropriate pretrained model with the default settings, proper hyperparameter tuning was performed to derive the final CNN model for infarction localization, and the derived model was validated through k-fold cross validation.

CNN model tunings were performed including the addition of three batch normalization layers, where one was before the flatten layer and the other two were after each dense layer, which was different from the standard VGG16 model (Supplementary Information S1: Default architecture of VGG16). The number of neurons was modified to 500 (first dense layer) and 250 (second dense layer) different from the standard 4,096. The output layer activation function was modified to Sigmoid from the default Softmax activation function for binary classification. So, the model could perform optimally when the feature difference among the inputs was complicated, and the feature differentiation between the infarction and non-infarction patches was challenging^37^. To adjust the learning rate adaptively with lower requirements of hardware and computational resources, Adam optimizer was used^38^. For loss minimization, Categorical Crossentropy loss function was considered as it performed well for the binary class where the inputs were encoded in the form of one-hot vector like (1, 0) for infarction and (0,1) for normal patches, respectively^39^.

To establish a robust infarction localization model, rigorous hyperparameter tuning was performed using the concept of random search technique as it outperforms the traditional grid search technique^40^. After performing several trails of experiments with different combinations of hyperparameters, a fine-tuned model was obtained by setting the optimal values such as learning rate = 0.001, batch size = 8, number of epochs = 4, number of steps per epoch = 5000 and dropout rate = 0.40 (first dropout layer) and 0.30 (second dropout layer).

In the k-folds cross validation strategy, to assess the robustness of the tuned-VGG16 CNN model as well as to handle the overfitting issue, the whole dataset of patches generated from 517 infarction patients were divided patient-wise into k-folds (k = 20) randomly. In each fold, the patches from 25 patients (5% of 517 patients) were selected randomly for testing; whereas the other 492 (95% of 517 patients) early infarction patients' data (patches) were used for training and validation purposes. The primary reason to consider k = 20 folds was to provide a larger set of training data to the machine in each round, so that the model could extract multiple distinct features, which could help correct recognition of unseen testing data. Finally, the best checkpoint model with the smallest validation loss and the highest average performance value was saved as the final derived model.

All implementations were carried out using the GPU version of TensorFlow 1.14 with the specification TITAN RTX 24GB × 4, Intel®Xeon®Scalable Processors, 3 UPI up to 10.4GT/s with 256 GB memory, Nvidia-smi 430.40 in Ubuntu 18.04.3 platform. Various predefined libraries such as Keras = 2:1:6, python = 3:6:9, numpy = 1:18:4, matplotlib = 3:2:1, OpenCV = 4:1, pillow = 7:1:2, and Scikit-learn = 0:21:3 were used in the image analysis.

### Infarction localization phase

The localization of classified abnormal (infarction) patches was performed on the respective NCCT using template matching algorithm developed by OpenCV (https://docs.opencv.org/4.x/d4/dc6/tutorial_py_template_matching.html). The designed localization system took the classified abnormal patches and the preprocessed NCCT altogether as the input, and matched those abnormal patches with the corresponding NCCT using the derived algorithm (Supplementary Information S1: Infarction localization phase).

### Statistical analysis

When performing the analysis of acute infarction patients using deep learning, the accuracy = (TP + TN)/(TP + FP + TN + FN) achieved by the models was not sufficient to evaluate the performance. Therefore, other performance metrics such as sensitivity/recall = TP/(TP + FN), specificity = TN/(TN + FP), precision = TP/(TP + FP), F-score = (2 × precision × sensitivity)/(precision + sensitivity), were used for evaluating the developed classification model. In the proposed model, the TP (true positives) represented the actual infarction patches predicted to be infarction as per requirement, and the TN (true negatives) denoted the non-infarction patches correctly predicted as non-infarction. Similarly, FP (false positives) predicted non-infarction as infarction, and FN (false negatives) incorrectly predicted the infarction as non-infarction. Apart from those performance metrics, the receiver operating characteristic (ROC) was also plotted to show the area under the curve (AUC) to predict the binary outcome. Average precision (AP) curve was also depicted to represent the trade-off between sensitivity and precision, which is useful in unbalanced dataset (https://scikit-learn.org/stable/modules/generated/sklearn.metrics.average_precision_score.html).

The model performance was also evaluated to compare the outcome of the patch-level accuracy = T_cp_/T_co_ and patient-level accuracy = T_cc_/T_p_. Where T_cp_ was the total number of correctly classified patches, T_co_ represented the total number of patches considered from both hemispheres during the infarction localization for individual patient, T_cc_ defined the total number that correctly identified patients with infarction lesion, and T_p_ was the total number of considered infarction patients.

## Results

### Patient demographics

Among the 9,353 patients screened, 517 (5.52%) met the inclusion criteria and were used for analysis. In these 517 patients, 355 had stroke onset time < 6 h, and 162 had stroke onset time between 6 and 12 h (Fig. 1). Patients were divided based on the infarction regions including supratentorial region (n = 428) and infratentorial region (n = 89). Supratentorial region comprised ACA, MCA and PCA areas which were further categorized into cortical (n = 156), subcortical (n = 204), and cortical plus subcortical (n = 68) areas. Similarly, infratentorial region comprised midbrain, pons, medulla and cerebellum. The current study also considered the analysis of infarction size 0.5–1.5 cm (n = 64) for both supratentorial and infratentorial regions. The clinical profiles of considered ischemic patients were represented in Table 1.

**Table 1:** Clinical profiles of the ischemic stroke patients recruited with onset time ≤ 6 h (h) and 6–12 h.

	≤ 12 h (n = 517)	≤ 6 h (n = 355)	6-12 h (n = 162)	P value ≤ 6 h vs. 6-12 h
Age (mean ± SD)	68.2 ± 12.7	68.1 ± 12.5	68.5 ± 13.2	0.690
Male sex, no. (%)	303 (58.6%)	222 (62.5%)	81 (50.0%)	0.007
Hypertension, no (%)	419 (81.0%)	287 (80.8%)	132 (81.4%)	0.864
Diabetes, no. (%)	201 (38.8%)	137 (38.5%)	64 (39.5%)	0.843
Hyperlipidemia, no. (%)	234 (45.2%)	153 (43.0%)	81 (50.0%)	0.143
Heart disease, no. (%)	186 (35.9%)	138 (38.8%)	48 (29.6%)	0.042
Old cerebral infarction, no. (%)	171 (33.07%)	114 (32.11%)	57 (35.18%)	0.491
Intracranial artery stenosis, no. (%)	365 (70.59%)	234 (65.91%)	124 (76.54%)	0.015

Statistics: Student's t-test for numerical data and Chi-square test for categorical data.

### CNN model and input size selection

The selection of the preferable patch size and the robust pretrained CNN model were carried out through several performance metrics (Table 2). For model selection, the primary metric AP was considered. Among all the models, the AP value of VGG16 for the patch size 140 × 140 was 0.69 which was higher than other pretrained models and patch sizes (Table 2 and Supplementary Fig. S6). Although, IR-v2 performed better (AP = 0.68) than VGG16 (AP = 0.55) for patch size 224 × 224, the other performance metrics like specificity = 0.70 and F-score = 0.68 were higher in the case of VGG16 (Table 2). Based on the results of the performance metrics (Table 2 and Supplementary Fig. S6), the pre-trained VGG16 model with input patch size 140 × 140 was selected for our CNN model to classify the infarction and non-infarction patches accurately.

**Table 2:** Performance metrics related to CNN model and patch size selection.

Patch sizes	Architectures	Accuracy	Sensitivity	Specificity	Precision	F-score	AUC	AP
24 × 24	GoogleNet	0.66	0.65	0.67	0.73	0.68	0.65	0.36
	Inception-v3	0.67	0.66	0.68	0.73	0.69	0.67	0.62
	Inception-v4	0.65	0.65	0.67	0.73	0.68	0.68	0.52
	VGG16	0.65	0.61	0.72	0.79	0.69	0.67	0.62
	ResNet50	0.65	0.63	0.68	0.77	0.69	0.67	0.60
	IR-v2	0.66	0.65	0.55	0.68	0.66	0.67	0.61
64 × 64	GoogleNet	0.63	0.59	0.72	0.81	0.68	0.66	0.44
	Inception-v3	0.66	0.63	0.71	0.74	0.68	0.67	0.54
	Inception-v4	0.66	0.63	0.69	0.72	0.67	0.67	0.56
	VGG16	0.66	0.66	0.66	0.63	0.64	0.67	0.64
	ResNet50	0.67	0.66	0.67	0.65	0.66	0.67	0.63
	IR-v2	0.66	0.64	0.68	0.67	0.66	0.67	0.60
140 × 140	GoogleNet	0.59	0.55	0.77	0.90	0.68	0.66	0.29
	Inception-v3	0.66	0.67	0.65	0.59	0.63	0.67	0.68
	Inception-v4	0.67	0.65	0.68	0.68	0.66	0.67	0.60
	VGG16	0.66	0.67	0.64	0.57	0.62	0.66	0.69
	ResNet50	0.66	0.62	0.71	0.75	0.68	0.67	0.53
	IR-v2	0.66	0.62	0.72	0.77	0.69	0.67	0.52
224 × 224	GoogleNet	0.64	0.60	0.73	0.89	0.69	0.67	0.45
	Inception-v3	0.66	0.67	0.65	0.59	0.63	0.67	0.62
	Inception-v4	0.66	0.62	0.71	0.75	0.68	0.67	0.53
	VGG16	0.66	0.63	0.70	0.74	0.68	0.67	0.55
	ResNet50	0.66	0.62	0.73	0.78	0.69	0.68	0.51
	IR-v2	0.66	0.67	0.65	0.58	0.62	0.67	0.68
299 × 299	GoogleNet	0.62	0.58	0.73	0.83	0.68	0.66	0.40
	Inception-v3	0.65	0.69	0.63	0.52	0.59	0.66	0.50
	Inception-v4	0.66	0.65	0.68	0.67	0.66	0.67	0.60
	VGG16	0.66	0.63	0.72	0.75	0.69	0.68	0.66
	ResNet50	0.65	0.61	0.73	0.79	0.69	0.67	0.48
	IR-v2	0.63	0.58	0.74	0.84	0.69	0.66	0.40

*AUC* area under curve, *AP* average precision, *VGG16* visual geometry group 16, *ResNet50* residual networks 50, *IR-v2* inception-ResNet-v2.

### CNN model finalization

The average testing values obtained by using 20-folds of the experiment were considered. The results of different performance metrics with the corresponding mean, obtained after performing a 20-fold cross-validation study, are presented in Table 3.

**Table 3:** Performance evaluation of the tuned-VGG16 infarction detection model.

Stroke onset time	# of patients	Accuracy	Sensitivity	Specificity	Precision	F-score	AUC		AP
Total patient-wise analysis									
≤ 6 h	355	0.72	0.66	0.78	0.77	0.65	0.73	0.69	
6–12 h	162	0.73	0.68	0.79	0.78	0.68	0.74	0.70	
Mean	–	0.73	0.67	0.78	0.77	0.66	0.73	0.69	
Supratentorial region-wise analysis									
≤ 6 h	297	0.71	0.72	0.69	0.74	0.68	0.71	0.70	
6–12 h	131	0.75	0.82	0.67	0.78	0.76	0.75	0.76	
Mean	–	0.73	0.77	0.68	0.76	0.72	0.73	0.73	
Infratentorial region-wise analysis									
≤ 6 h	58	0.74	0.60	0.89	0.81	0.63	0.74	0.69	
6–12 h	31	0.72	0.55	0.89	0.76	0.60	0.73	0.63	
Mean	–	0.73	0.57	0.89	0.78	0.61	0.73	0.66	
Cortical area-wise analysis									
≤ 6 h	119	0.69	0.65	0.73	0.76	0.63	0.69	0.73	
6–12 h	37	0.72	0.72	0.72	0.80	0.71	0.73	0.77	
Mean	–	0.70	0.68	0.72	0.78	0.67	0.71	0.75	
Subcortical area-wise analysis									
≤ 6 h	132	0.75	0.79	0.68	0.73	0.72	0.77	0.72	
6–12 h	72	0.78	0.88	0.65	0.77	0.79	0.78	0.78	
Mean	–	0.76	0.83	0.66	0.75	0.75	0.77	0.75	
Cortical plus subcortical area-wise analysis									
≤ 6 h	48	0.67	0.70	0.64	0.74	0.66	0.69	0.70	
6–12 h	20	0.73	0.80	0.66	0.76	0.74	0.74	0.74	
Mean	–	0.70	0.75	0.65	0.75	0.70	0.71	0.72	
Infarction size (0.5–1.5 cm)-wise analysis									
≤ 6 h	39	0.79	0.74	0.82	0.75	0.71	0.78	0.73	
6–12 h	25	0.78	0.81	0.75	0.79	0.76	0.78	0.78	
Mean	–	0.78	0.77	0.78	0.77	0.73	0.78	0.75	

*–* not mentioned, *AUC* area under curve, *AP* average precision.

**Figure 2:**
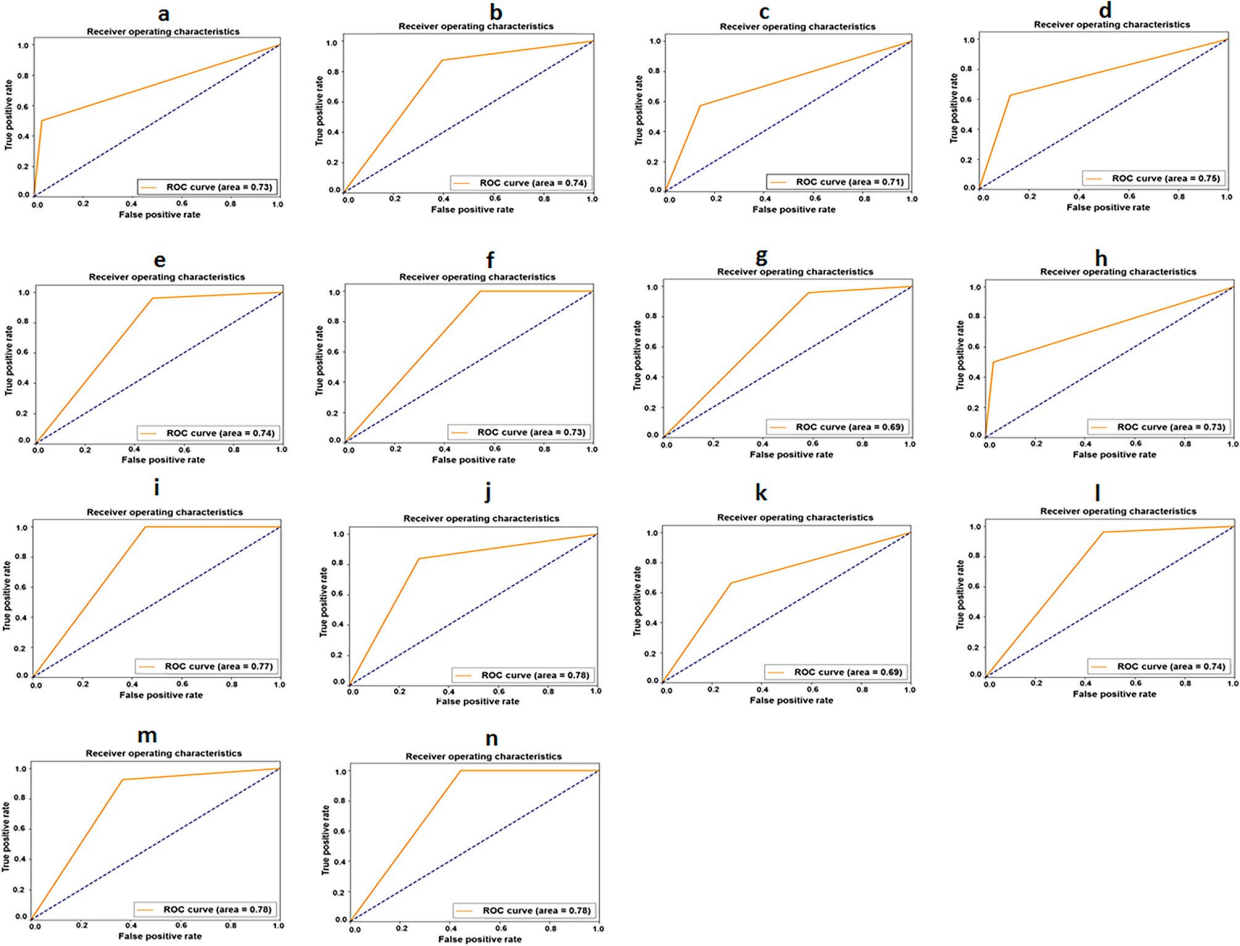
Receiver operating characteristics (ROC) curves generated from tuned-VGG16 infarction localization model. (**a**) Stroke onset time (≤ 6 h). (**b**) Stroke onset time (6–12 h). (**c**) Supratentorial region infarction (≤ 6 h). (**d**) Supratentorial region infarction (6–12 h). (**e**) Infratentorial region infarction (≤ 6 h). (**f**) Infratentorial region infarction (6–12 h). (**g**) Cortical area infarction (≤ 6 h). (**h**) Cortical area infarction (6–12 h). (**i**) Subcortical area infarction (≤ 6 h). (**j**) Subcortical area infarction (6–12 h). (**k**) Cortical plus subcortical area infarction (≤ 6 h). (**l**) Cortical plus subcortical area infarction (6–12 h). (**m**) Infarction size 0.5–1.5 cm (≤ 6 h). (**n**) Infarction size 0.5–1.5 cm (6–12 h). *ROC* receiver operating characteristic, *VGG16* visual geometry group 16.

The tuned-VGG16 model achieved the mean AUC = 0.73 (Table 3: 5th row and 8th column) along with mean specificity = 0.78 (Table 3: 5th row and 5th column) and precision = 0.77 (Table 3: 5th row and 6th column), respectively. The delineation of ROC curve showing individual AUC = 0.73 for stroke onset time ≤ 6 h and AUC = 0.74 for stroke onset time within 6–12 h (Fig. 2a,b) justified the uniformity of the derived model in infarction localization irrespective of the onset time. The localization of the infarction in the infratentorial region (Fig. 2c–f and Table 3: 11th, 12th rows and 8th column) showed the tuned-VGG16 model performed equivalently as supratentorial with AUC = 0.74 for stroke onset time ≤ 6 h and AUC = 0.73 for stroke onset time within 6–12 h. The mean specificity = 0.89 and mean precision = 0.78 (Table 3: 13th row and 5th, 6th columns) suggested the ability of the proposed CNN model to recognize the non-infarction patches (TNs) more precisely with less false positives (FPs). Further, the achievement of AP = 0.69 for stroke onset time ≤ 6 h and AP = 0.63 for stroke onset within 6–12 h signified the balanced outcome of higher precision and lower recall (Table 3: 11th, 12th rows and 9th column). In case of the supratentorial infarction, the derived localization model could correctly determine the TP (infarction region) with the mean value of AUC = 0.73 (Table 3: 9th row and 8th column) and sensitivity = 0.77 (Table 3: 9th row and 4th column).

Considering the analysis of infarction in cortical area, the developed model achieved mean AP = 0.75 (Table 3: 17th row and 9th column) with AUC = 0.69 for stroke onset ≤ 6 h and AUC = 0.73 for stroke onset time within 6–12 h (Fig. 2g,h). Further, the F-score = 0.72 for stroke onset time ≤ 6 h and 0.79 for stroke onset time within 6–12 h in subcortical infarction (Table 3: 19th, 20th rows and 7th column) signified the harmonic balance between higher recall and lower precision. For the cortical plus subcortical infarction, the model achieved a significant outcome with sensitivity and AP ≥ 0.70 for both stroke onset time (Table 3: 23rd, 24th rows and 4th, 9th column). As presented in Fig. 2i–l, the ROC curve showing the AUC = 0.77 (stroke onset time ≤ 6 h) and AUC = 0.78 (stroke onset time 6–12 h) in the cases of subcortical infarction along with the value of AUC = 0.69 (stroke onset time ≤ 6 h) and AUC = 0.74 (stroke onset time 6–12 h) for cortical plus subcortical infarction signified the ability of tuned-VGG16 model in differentiation between all positives (TP, TN) and negatives (FP, FN).

The derived model achieved AUC = 0.78 for both stroke onset time in the cases of infarction size $\le$ 1.5 cm (Fig. 2m,n) with mean sensitivity = 0.77 and AP = 0.75 (Table 3: 29th rows and 4th, 9th column), conveying the stability of the selected CNN model for the localization of small infarction.

**Figure 3:**
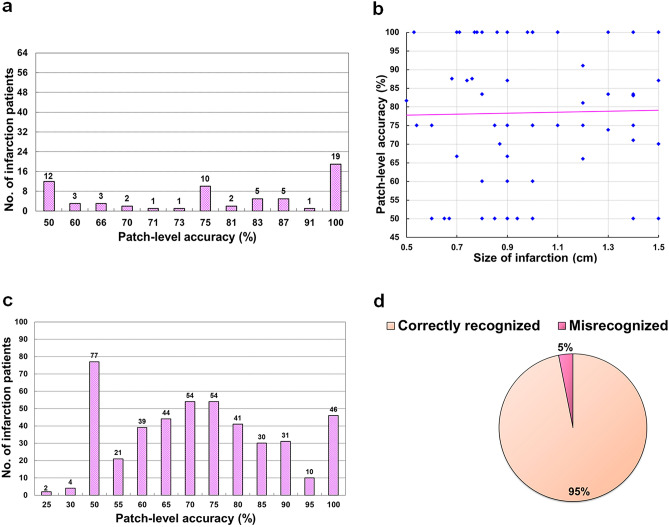
The analysis of patch-level and patient-level accuracy. (**a**) Analysis of patch-level accuracy (%) for patients with infarction size ≤ 1.5 cm. (**b**) Analysis of patch-level accuracy (%) for patients with infarction size 0.5–1.5 cm. (**c**) Analysis of patch-level accuracy (%) for patients with infarction size > 1.5 cm. (**d**) Analysis of patient-level accuracy.

Considering the patch-level accuracy, the tuned-VGG16 model achieved 100% accuracy without any misclassified infarction patch in 19 out of 64 patients for the infarction size ≤ 1.5 cm and 46 out of 453 patients for the infarction size > 1.5 cm (Fig. 3a,c). The accuracy varied from 60 to 100% in the 23 patients with even a smaller infarction size ≤ 0.9 cm (scatter plot in Fig. 3b). In the case of infarction size > 1.5 cm (Fig. 3c), the tuned-VGG16 achieved patch-level accuracy ≥ 70% for 266 out of total 453 stroke patients.

The patient-level accuracy analysis using the tuned-VGG16 model showed the derived VGG16 model could correctly recognize 494 out of 517 patients (95%, Fig. 3d) even for those patients with a single classified infarction patch (TP).

### Infarction localization on NCCT

**Figure 4:**
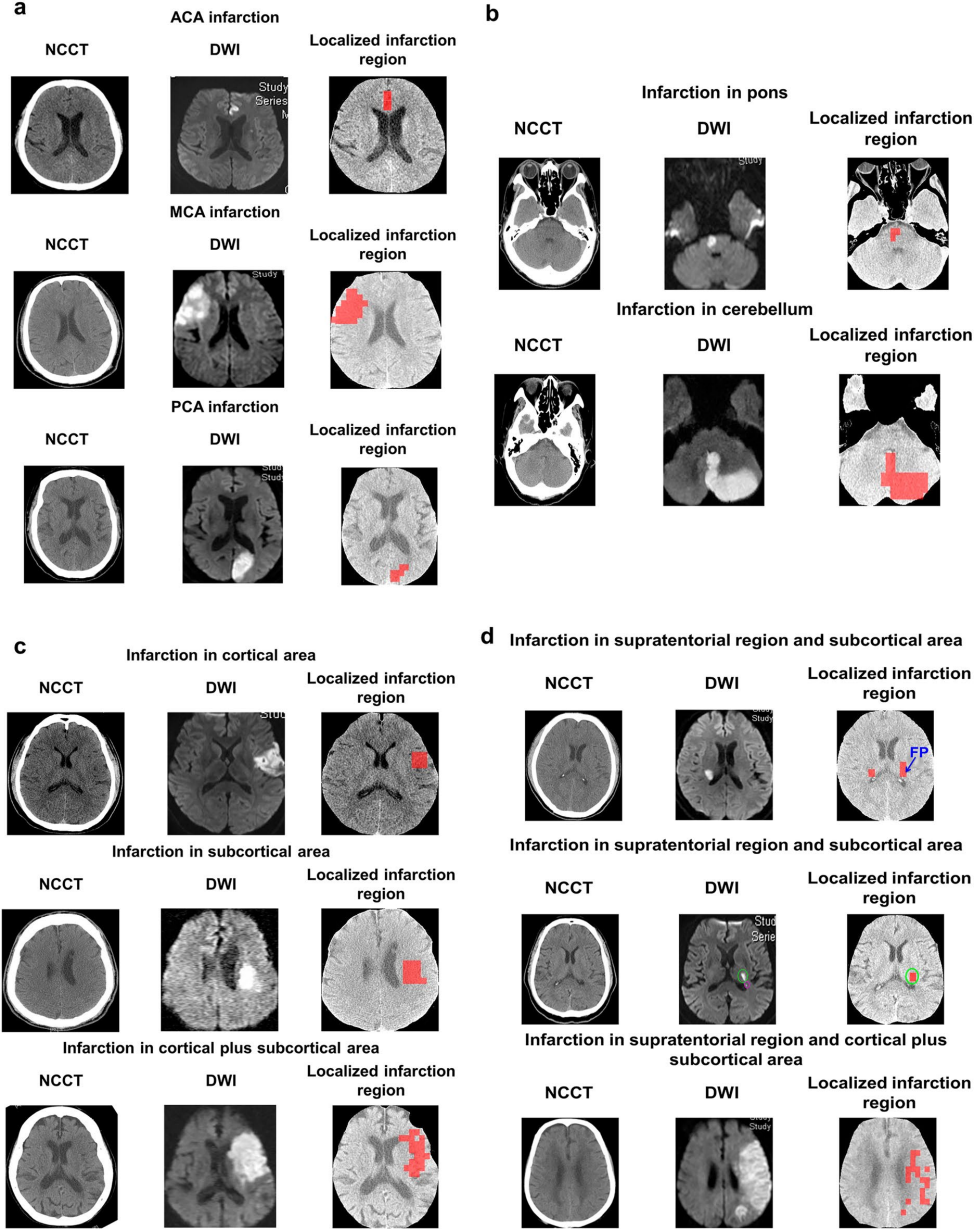
Localization of early infarction on first-line NCCT. (**a**) Automatic localization of early infarction in supratentorial region. (**b**) Automatic localization of early infarction in infratentorial region. (**c**) Automatic localization of early infarction in cortical, subcortical and cortical plus subcortical areas. (**d**) Inaccurate localization of infarction. It could be observed that the tuned-VGG16 model incorrectly localized the infarction in the opposite hemisphere, which was FP (3rd row). Further, there were two distinct infarctions located in the DWI (6th row) represented by the green and purple circles, respectively. In these cases the developed model could accurately localize the bigger size of the infarction on NCCT (green circle), whereas failed to identify the comparatively smaller one. Besides, it could be visualized from the localized NCCT slice (9th row), that the identified infarction region was smaller than the corresponding DWI, where some of the ischemic patches were misclassified as normal (FNs). *NCCT* non-contrast computed tomogram, *DWI* diffusion-weighted imaging.

The infarction localization model was developed to automatically display the infarction region on the corresponding NCCT (Fig. 4 and Supplementary Fig. S7). As shown in Fig. 4, the finalized tuned-VGG16 localization model could successfully recognize the abnormal patches in both supratentorial and infratentorial brain regions (Fig. 4a,b and Supplementary Fig. S7a) and also in cortical, subcortical and cortical plus subcortical areas (Fig. 4c and Supplementary Fig. S7b).

Although the infarction localization model could correctly identify the patches of different infarction size in the corresponding NCCTs, there were some cases where the localized infarction in NCCT (Fig. 4d) was smaller than the DWI/ADC (FNs). In some instances, the tuned-VGG16 model localized the infarction on the normal region of the opposite hemisphere (Fig. 4d) by misclassifying the non-infarction patches as infarction (FPs). However, this type of wrong localization could be managed by the clinicians considering the neurological deficit criteria.

## Discussion

Our previous study^3^ developed a CNN-based model to identify the early ischemic injury on the first-line NCCT, which could accurately classify the normal and ischemic stroke patients by identifying the probable ischemic slices. However, the previous study has the limitation to localize the infarction on these NCCT slices to know the region, size, and severity of the infarction^15–25,41,44^. The present study was reformed to develop a supervised deep learning (DL)-based localization model for early infarction sign by integrating several automatic methods and software. The proposed model is considering not only the stroke onset time < 12 h, but also the different regions (supratentorial, infratentorial) and areas (cortical, subcortical, cortical plus subcortical), and even the lacune-size infarction. Although few related works developed DL-based infarction localization model using NCCT^15–23,41,44^, none of these image analyses were performed in both infratentorial and supratentorial regions considering the complicacy of localization in cortical and subcortical areas. A detailed comparison of those related studies related to clinical contribution was presented in Table 4.

**Table 4:** Comparison of the different IS localization models using NCCT.

Comparison parameters	Qiu et al.^20^	Chin et al.^19^	Nishio et al.^18^	Beecy et al.^41^	Kuan et al.^17^	Pan et al.^15^	Our work
Goal	Detection and quantification of IS	Detection of early IS	Detection of acute IS	Diagnosis of acute infarction	Segmentation of early infarct	Identify ischemic lesions	Detection and localization of early IS
Image type	NCCT	NCCT	NCCT	NCCT	NCCT	NCCT	NCCT
Stroke onset time	< 8 h	–	–	–	–	< 9 h	< 12 h
Brain part	M1	–	–	–	–	–	Whole brain
Brain region	–	–	–	–	–	–	Supratentorial, Infratentorial
Brain area	–	–	–	–	–	Anterior and posterior area	Cortical, subcortical, cortical plus subcortical
Infarction size	–	–	–	–	–	–	≥ 0.5 cm
No. of IS patients	257	–	238	114	260	116	517
Accuracy	–	0.92	–	0.88	0.85	0.74	0.73 (mean)
Sensitivity	–	–	0.41	0.65	–	–	0.67 (mean)
Specificity	–	–	–	0.91	–	–	0.78 (mean)
Precision	–	–	0.62	–	–	–	0.77 (mean)
F-score	–	–	0.49	–	0.44	–	0.66 (mean)
AUC	–	–	–	0.91	0.83	–	0.73 (mean)
AP	–	–	–	–	–	–	0.69 (mean)
Limitations of the related works	1. Only stroke in M1 segment is considered 2. No performance analysis related to IS localization outcome 3. Single hospital study 4. Same scanner and image acquisition protocols of NCCT	1. Limited study population 2. No contribution towards localization	1. Sensitivity ≈ 40%, precision ≈ 60%, F-score < 50% 2. Clear visibility of infarction lesion on NCCT	1. Limited study population 2. Visible stroke lesions on NCCT 3. High imbalance data This is one of the main reasons for low sensitivity 4. Single hospital study	1. Only MCA region is considered 2. Low F-score value = 0.44	1. Limited study population 2. Visible infarction lesion on NCCT 3. Lack of external validation	1. Limitation to localize if the infarction size < 0.5 cm 2. Sometimes, the old and new infarction both localized together 3. Generation of FPs during whole slide localization 4. Single hospital study 5. Same scanner and image acquisition protocols of NCCT

*–* not mentioned, *NCCT* non-contrast computed tomogram, *MCA* middle cerebral artery, *AUC* area under curve, *AP* average precision, *IS* ischemic stroke.

Although, the previously proposed works used first-line NCCT and DL methodologies for early ischemic stroke detection and segmentation, several technical limitations exist in terms of model development, data partition and performance evaluation^15,17,18,41–44^. For instance, most of the previous works performed slice-wise analysis^17,18,41,43,44^, where the global features generated from other cerebral parts like sulcus, artery, and ventricle dominate the local features of infarction, resulting higher FNs. Therefore, the sensitivity (0.41^18^, 0.65^41^) and F-score value (0.44^17^, 0.49^18^) of those related works are very less. In contrast, we trained the model by providing both local and global information in the form ischemic and normal patches through our patch-based solution extracted from the opposite hemisphere.

The patch-based analysis was performed by using first-line NCCT^15,42^. In the first-work^15^, ResNet used for patch classification considering the sizes 17 × 17, 19 × 19 and 23 × 23, whereas in the second work^23^ two-stage model (Unet-ResNet) was employed for the early ischemic stroke segmentation by considering a fixed patch size of 23 × 23. The smaller size of patches might lead to feature distortion while performing the internal resizing^43,44^ while input to the ResNet whose default size is 224 × 224. This might lead to inadequate feature extraction, especially when the infarction size was so large or too small. Accordingly, we used the patch of size of 140 × 140 for qualitative feature extraction after an extensive performance analysis of different patch sizes as presented in Table 2. The adopted size of patches enables the network to differentiate both infarction and normal regions effectively^45,46^. Besides, the execution of sequential VGG16 is faster than the two stages UNet-ResNet^23^, where the computational-intensive segmentation was performed first following the classification.

In our current study, a large study population of 517 patients with stroke onset < 12 h considered while the previous works were developed on limited datasets^15,43^. For instance, there were only 116 patients recruited for patch-wise classification^15^. The precise segmentation of early infarction using multi-scale U-Net was developed by considering only 30 ischemic cases^43^. Further, the primary reason of low sensitivity (0.65) in the DL-assisted IS detection model^41^ could be imbalance data (normal slice: 6076, ischemic slice: 732). Therefore, the model could learn the normal features (TNs) only. Our proposed model was developed by considering the NCCT slices without visibility of ischemic lesion for stroke onset < 12 h, where the differentiation of ischemic and normal features was challenging. On contrary, there was a clear visibility of the stroke region in the prior proposed works^18,41–43^ for the easy differentiation.

One more novel point of our analysis is the automatic generation of ground truth for the AI analysis. In most of the related works^15,17,19,20,41,44^, the training images were needed to be annotated first, and the annotation procedure might contain some errors based on the doctor's experience and expertise. In some of the clinical scenarios, the infarction is not visible in early IS, where the annotation is difficult using the naked eye. Therefore, we constructed an automatic model combining the dual image modalities using both DWI/ADC and NCCT with the imaging interval less than 14 days to minimize the chances of annotated errors. The automatic model can not only reduce the burden of the clinicians but also increase the annotation accuracy as every procedure of annotation is performed automatically. In one of the studies^44^, the neurologists generated the ground truth for early ischemic stroke segmentation model without considering any reference image. Besides, there was no outcome validation. Hence, there might be potential diagnostic verification bias.

The reviewed dataset used for AI analysis was relatively comprehensive in comparison to the analysis performed in the previous related works^15–23,41–46^. Our rigorous study design achieved a mean accuracy = 0.73 for both stroke onset durations even with the variation of ischemic changes (Table 3: 5th row, 3rd column). The main reason of achieving lower mean sensitivity = 0.57 (Table 3: 13th row, 4th column) for infratentorial infarction in comparison to supratentorial infarction is likely due to the small number of considered patients (n = 89) and also the slower ischemic change resulting in the increase of FNs (the infarction patch misclassified as normal patch). The achievement of the patch-level classification accuracy ≥ 80% for 32 out of 64 patients with an infarction size 0.5–1.5 cm (Fig. 3a), inferred the effectiveness of the developed model in detecting lacunar infarction. Further, it could be observed from the pattern of the scatter plot’s trend-line (Fig. 3b) that the patch-level accuracy of the localization model could be increased along with an increase in the number of patients, which was one of the notable points of the derived model. Besides, the CNN model developed in the current study could classify patient-wise one or two patches for some participants (n = 83) with infarction size > 1.5 cm. Hence, the percentage of patch-level accuracy for those considered patients was ≤ 50% (Fig. 3c). However, in the case of early ischemic stroke, it is sufficient to know the location of the infarction even with a single correctly classified patch (TP).

There are some limitations in the present study. First, during the testing of whole NCCT slices, few FPs (normal patch misclassified as abnormal) were generated (Fig. 4d: 3rd row), especially for the patients with stroke onset time < 2 h, where the infarction is minute in comparison to contralateral side. However, this mistake could be overcome using the information of neurological deficit since supratentorial infarction may cause neurological abnormalities on the contralateral body and clinical information. Second, we found in some IS patients, the size of infarction region localized by patches is smaller than that in DWI/ADC (Fig. 4d: 6th and 9th rows) which signified that some abnormal patches were classified as normal. This is due to the extended time gap between the initial NCCT and the follow-up DWI/ADC, which might affect the infarction outcome. Third, it could be observed that the localization accuracy was higher in large infarctions than small infarctions. The reason might be the imbalanced distribution of healthy tissue and the infarction. Hence, during the model performance, the learned features from the normal region dominated the distinguished features of the infarction region. Further, the interval between the DWI/ADC and initial NCCT could be another potential reason. Therefore, the model achieved good accuracy to classify only when equal number between infarction and normal patches extracted from both hemispheres was given as input. However, the biasedness of the derived model could be observed by testing the patches generated from the whole NCCT slices. Consequently, either the infarction patches were misrecognized as non-infarction or the infarction was wrongly detected on the non-infarction regions due to the misclassification of the normal patches. Fourth, sometimes in the case of patients with both old and recent strokes, the infarction in the old stroke was detected instead of the recent stroke. Fifth, all the study images were collected from a single center, which may not be able to be generalized in other medical systems. The validation of the proposed automatic ischemic region localization system may be needed in other medical systems with different MR and NCCT sequences. Also, a prospective study collecting images in the emergency department will be the next aim of this study. Sixth, the improvement of our system to localize tiny infarct of size < 0.5 cm considering the features from whole NCCT slice is necessary.

## Conclusion

The present study set up an AI-based automatic model with the concept of automatic feature extractor using DL to detect early infarction sign in both supratentorial and infratentorial regions with stroke onset < 12 h and examine the different brain areas including cortical, subcortical and cortical plus subcortical and also infarction size 0.5–1.5 cm.

### Supplementary Information

Supplementary Information.

## Data Availability

The data used for the primary dataset, stroke code test sets and international test were obtained from hospitals as described above. Data use was approved by relevant institutional review boards. The datasets generated and/or analyzed during the current study are not publicly available due to privacy issues of the patients but are available from the corresponding author on reasonable.
