# Supplementary material for: Localization of early infarction on non-contrast CT images in acute ischemic stroke with deep learning approach
Source: Sci Rep. 2023 Nov 9;13:19442. doi: 10.1038/s41598-023-45573-7 (PMC10636036; doi:10.1038/s41598-023-45573-7)
Supplement: Supplementary file 1 — Supplementary Information. [file 41598_2023_45573_MOESM1_ESM.pdf]

Localization of Early Infarction on Non-Contrast CT Images in Acute Ischemic Stroke with Deep Learning Approach

Sulagna Mohapatra[1], Tsong-Hai Lee[2,3,†], Prasan Kumar Sahoo[1,2,*], Ching-Yi Wu[4]

[1] Department of Computer Science and Information Engineering, Chang Gung University, Taoyuan, Taiwan

[2] Department of Neurology, Linkou Chang Gung Memorial Hospital, Taoyuan, Taiwan

[3] College of Medicine, Chang Gung University, Taoyuan, Taiwan

[4] Department of Occupational Therapy & Graduate Institute of Behavioral Sciences, College of Medicine, Chang Gung University, Taoyuan, Taiwan

† Sulagna Mohapatra and Tsong-Hai Lee contributed equally to this work.

* Corresponding author

Prasan Kumar Sahoo, PhD

Department of Computer Science and Information Engineering, Chang Gung University, Taoyuan, Taiwan

Department of Neurology, Chang Gung Memorial Hospital, Linkou Medical Center, Taoyuan, Taiwan

259, Wen-Hwa 1st Road, Guishan, Taoyuan, 33302, Taiwan

TEL: +886-3-211-8800, ext. 3804

E-mail: pksahoo@mail.cgu.edu.tw

Supplementary Information

Supplementary Fig. S1

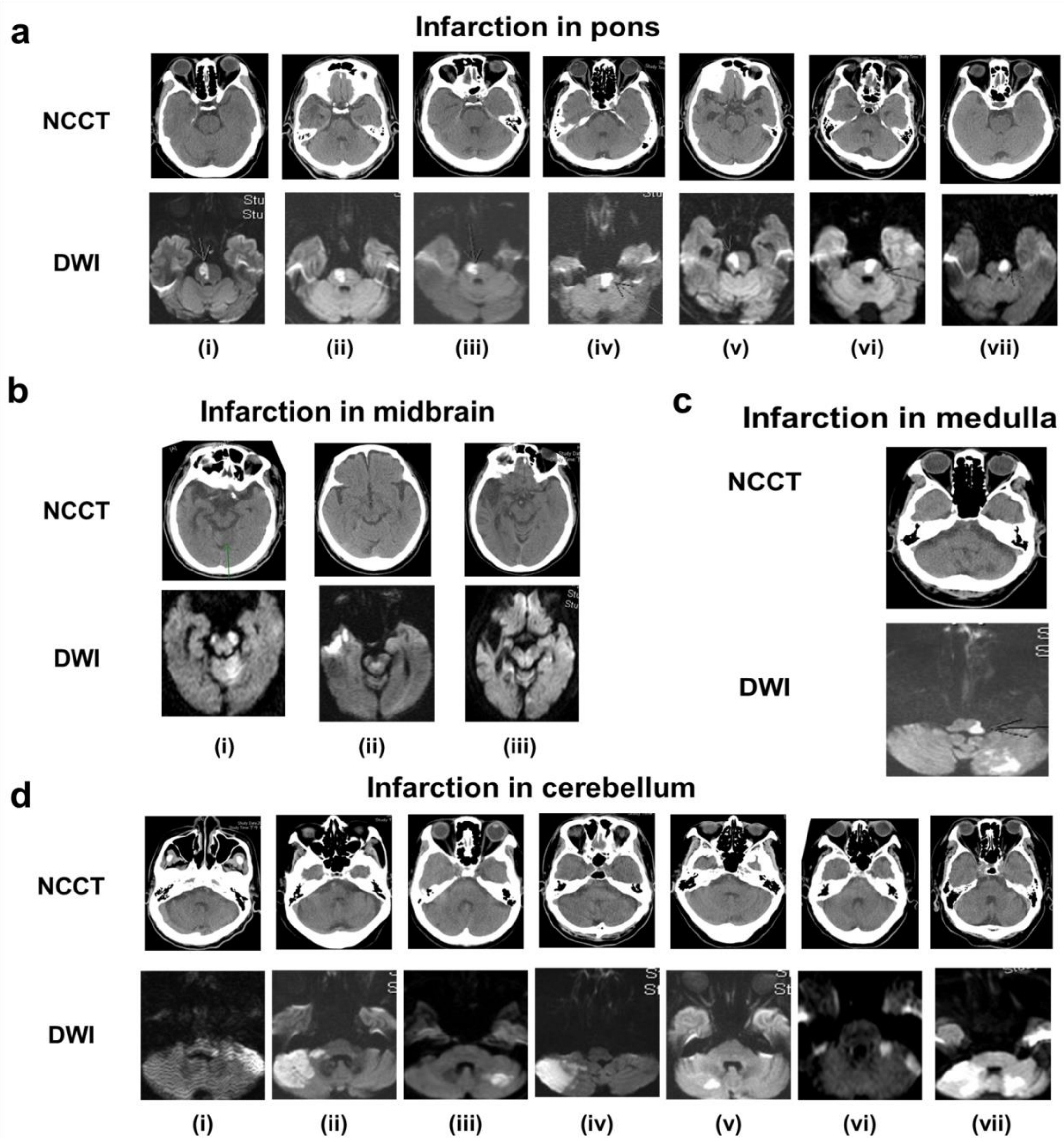

Infarction occurred in infratentorial region. (a) Infarction in pons. **(b)** Infarction in midbrain. **(c)** Infarction in medulla. **(d)** Infarction in cerebellum.

NCCT, non-contrast computed tomogram; DWI, diffusion-weighted imaging.

Supplementary Fig. S2

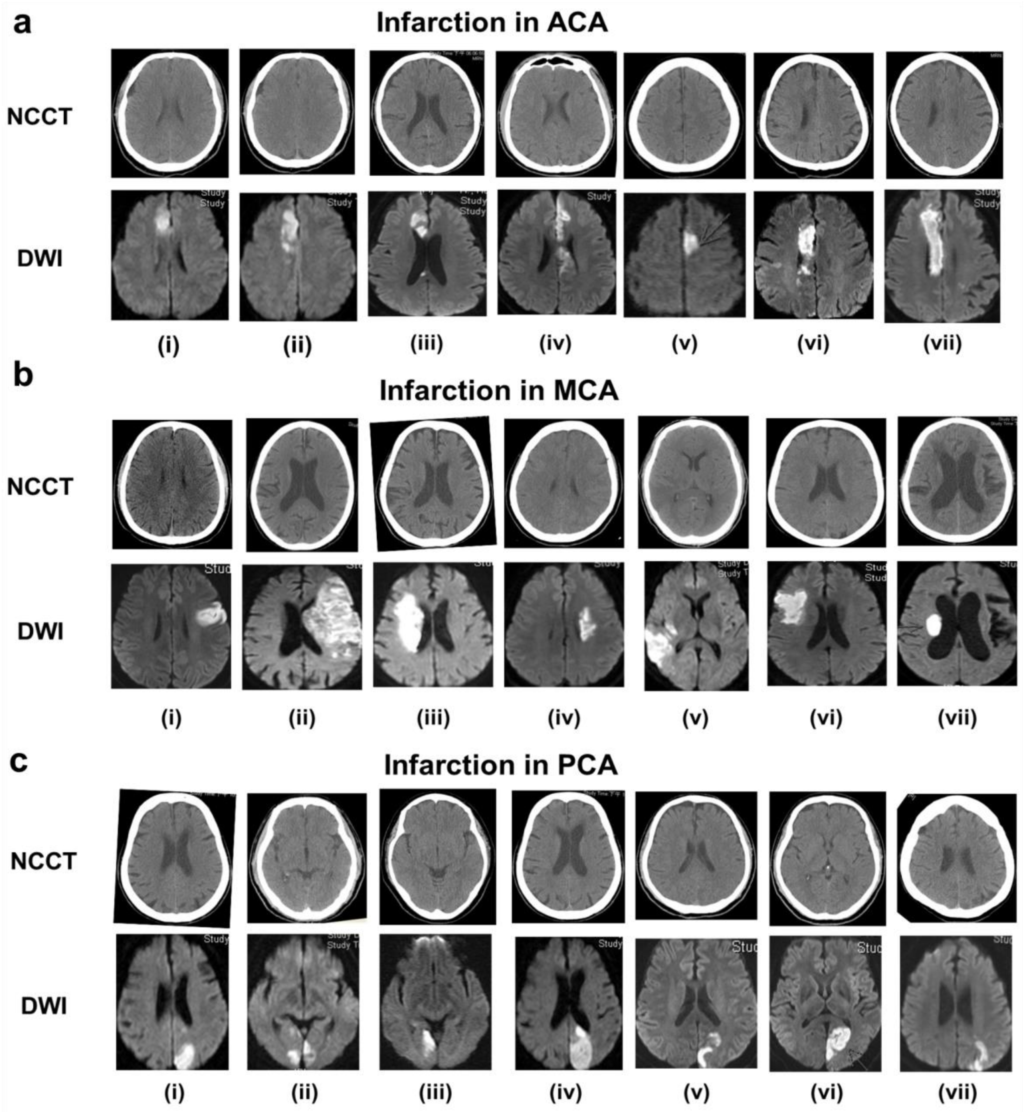

Infarction occurred in supratentorial region. **(a)** Infarction in ACA. **(b)** Infarction in MCA. **(c)** Infarction in PCA.

NCCT, non-contrast computed tomogram; DWI, diffusion-weighted imaging; ACA, anterior cerebral artery; MCA, middle cerebral artery; PCA, posterior cerebral artery.

Supplementary Fig. S3

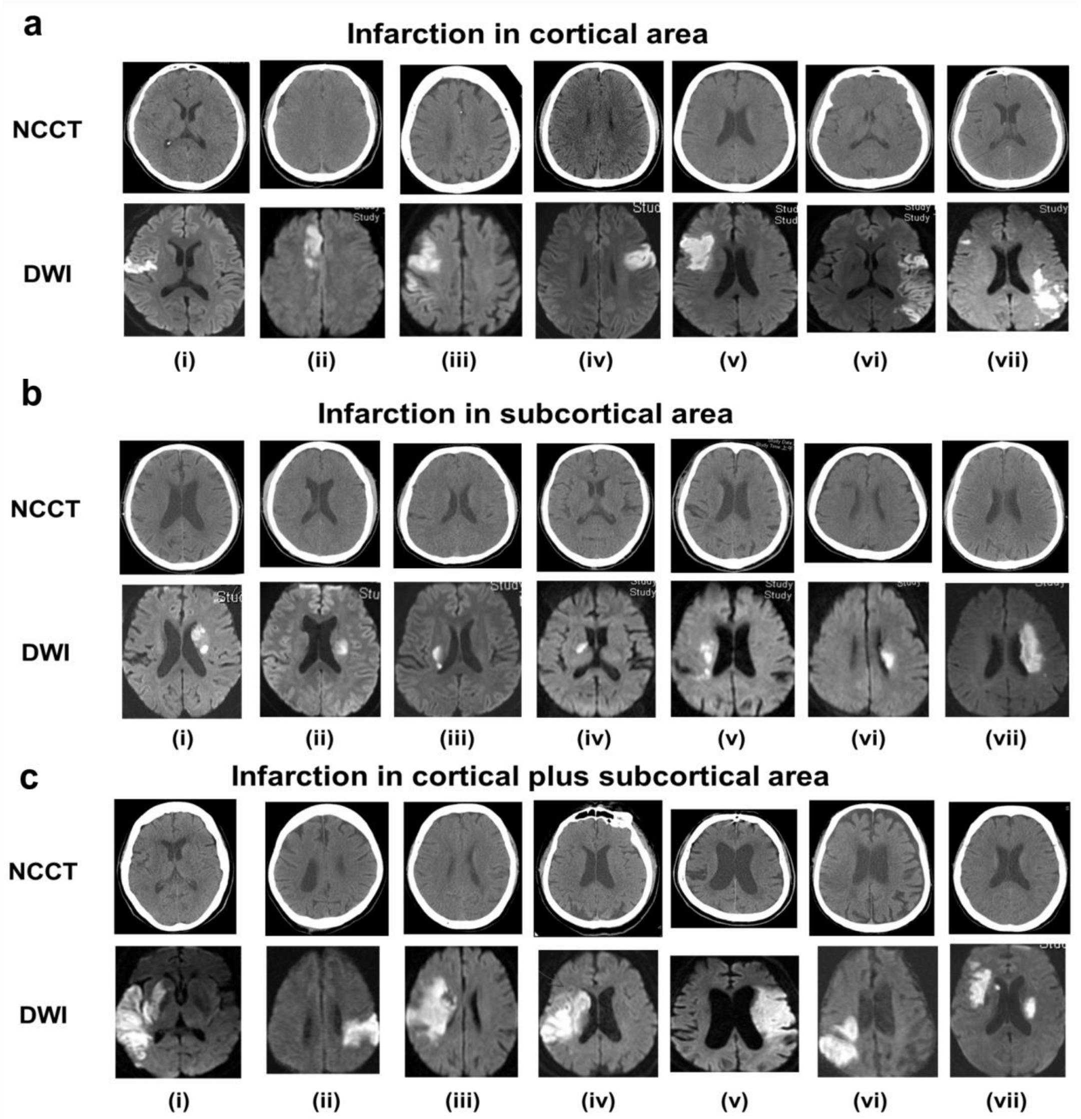

Infarction occurred in different brain areas. (a) Infarction in cortical area. **(b)** Infarction in subcortical area. **(c)** Infarction in cortical plus subcortical area.

NCCT, non-contrast computed tomogram; DWI, diffusion-weighted imaging.

Supplementary Fig. S4

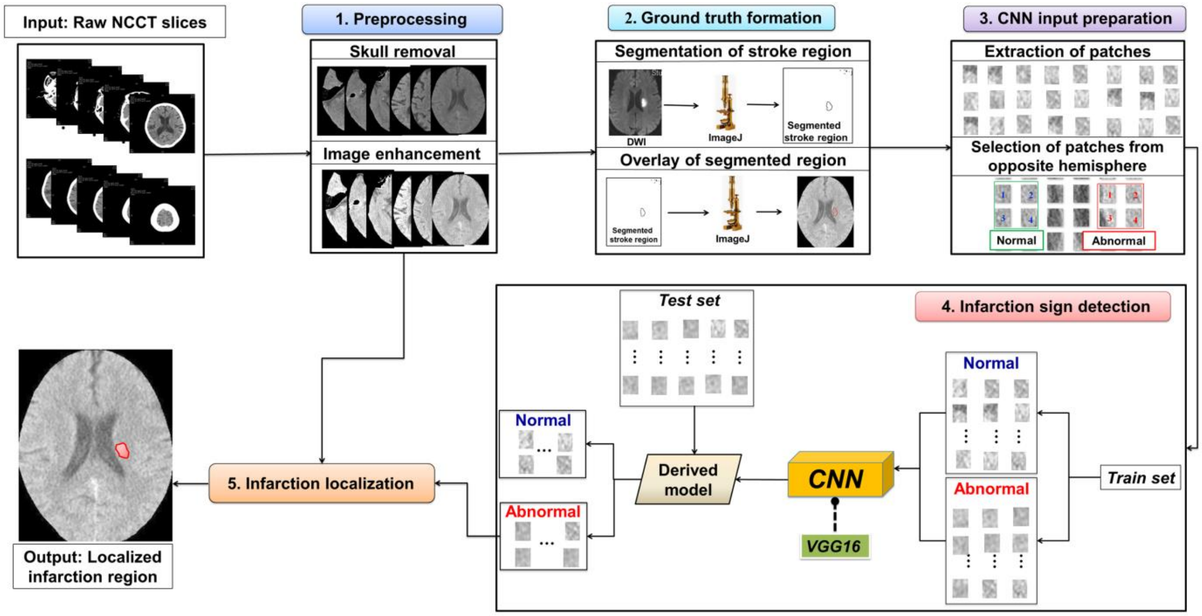

Overview of infarction localization model using deep learning.

NCCT, non-contrast computed tomogram; DWI, diffusion-weighted imaging; CNN, convolutional neural network; VGG16, Visual Geometry Group 16; ResNet50, Residual Networks 50; IR-v2, Inception-ResNet-v2.

Supplementary Fig. S5

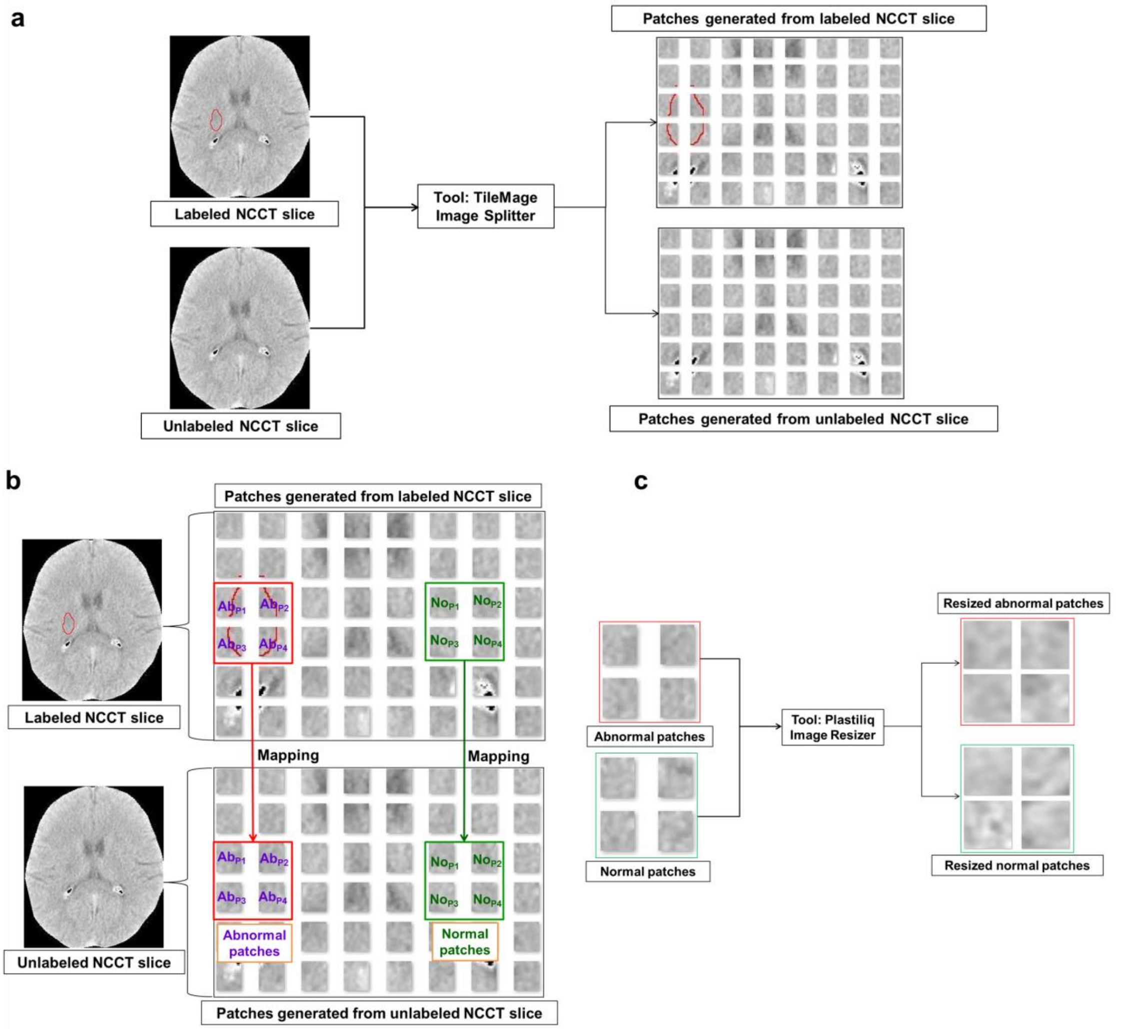

Different sub-phases of CNN input preparation phase. (a) Generation of normal and abnormal patches. **(b)** Selection of abnormal and normal patches. One of the highlighted points of the proposed idea was the way that we followed for selection of both normal and abnormal patches. The input patches were extracted from both annotated and unannotated NCCT slices. For the patch selection, first, the patches generated from the labeled NCCT slices were considered. The patches that fell within the annotation (Ab_{p1}, Ab_{p2}, Ab_{p3}, Ab_{p4}) were considered as abnormal patches. After that, the normal patches (No_{p1}, No_{p2}, No_{p3}, No_{p4}) were selected from the opposite hemisphere by following the

brain symmetricity rule. Once both the set of normal and abnormal patches were marked on the labeled slices, those patch numbers were mapped with the patches of the unlabeled slice. The mapped abnormal and normal patches on the unlabeled/unannotated NCCT slice were finally considered for the deep learning analysis. **(c)** Resizing of selected abnormal and normal patches. The necessity of the patch resizing is because of the following reasons. Firstly, if the image size is too small in comparison to the DL models on which it is trained, then the network might not read the important features properly affecting the accuracy. Similarly, increasing the image size too much might make the network skip some primary features or might consider the ROI as artifacts resulting in the decrease of accuracy with more loss. Therefore, it is important to select the suitable patch size for the input to the CNN models.

NCCT, non-contrast computed tomogram; DWI, diffusion-weighted imaging.

Default architecture of VGG16. The network is 16 layers deep CNN and takes RGB image of dimension 224 × 224 × 3 as input and uses the filter of size 3 × 3[1]. The VGG network consists of five blocks of convolutional layers, consisting of two convolution layers and one max-pooling layer in block 1 and block 2. This is followed by the sequential combination of 3 convolution layers and one max-pooling layer in blocks 3, 4, and 5. Although the numbers of convolution layers are the same in blocks 1 and 2 and the same in blocks 3, 4 and 5, the number of filters allowed in the convolution layers of each block differs. For instance, the numbers of kernels are 64, 128, 256, 512, 512 for block 1, block 2, block 3, block 4 and block 5, respectively. In addition to this, the stride for the convolution layers is fixed as 1 to preserve the spatial information after convolution. The 5 blocks are followed by three fully connected layers, which serve as a typical multi-layer perceptron. The first two fully connected layers consist of 4096 neurons each and the last fully connected layer consists of 1000 neurons, representing one class each. In addition to the layers, the activation function used in the convolution layer is ReLU and for the final layer, the activation function used is Softmax.

Infarction localization phase. To provide a complete solution and to assist the clinicians for easy-view of the ischemic region, the localization of classified abnormal patches on the respective NCCT was performed using template matching algorithm in OpenCV[a]. In the developed template matching algorithm, the patch as the template (T) to the corresponding source unlabeled NCCT (I) was provided using normalized correlation (CV TM CCOEFF NORMED) method. Based on the degree of correlation, the template was laid over the NCCT to localize the ischemic region. To identify the matching area, the template image (patch) needs to be moved against the source (NCCT) pixel by pixel in both horizontal and vertical directions. At each location, a metric was calculated to represent how similar the patch was to that particular area of the corresponding source NCCT and was defined by equation (1):

$$R(h,v) = \frac{\sum_{(\acute{h},\acute{v})}\left(T(h,\acute{v}).I(h+h,v+\acute{v})\right)}{\sqrt{\sum_{(\acute{h},\acute{v})}(T\left(\acute{h},\acute{v}\right)^{2}.\sum_{(\acute{h},\acute{v})} I(h+\acute{h},v+\acute{v})^{2})}} \qquad (1)$$

Where, R represents the resultant matrix that stores the metric of each location of T over I and (h, v) is considered as the horizontal and vertical location in R that contains the match metrics.

[a] https://docs.opencv.org/4.x/d4/dc6/tutorial_py_template_matching.html

Supplementary Fig. S6

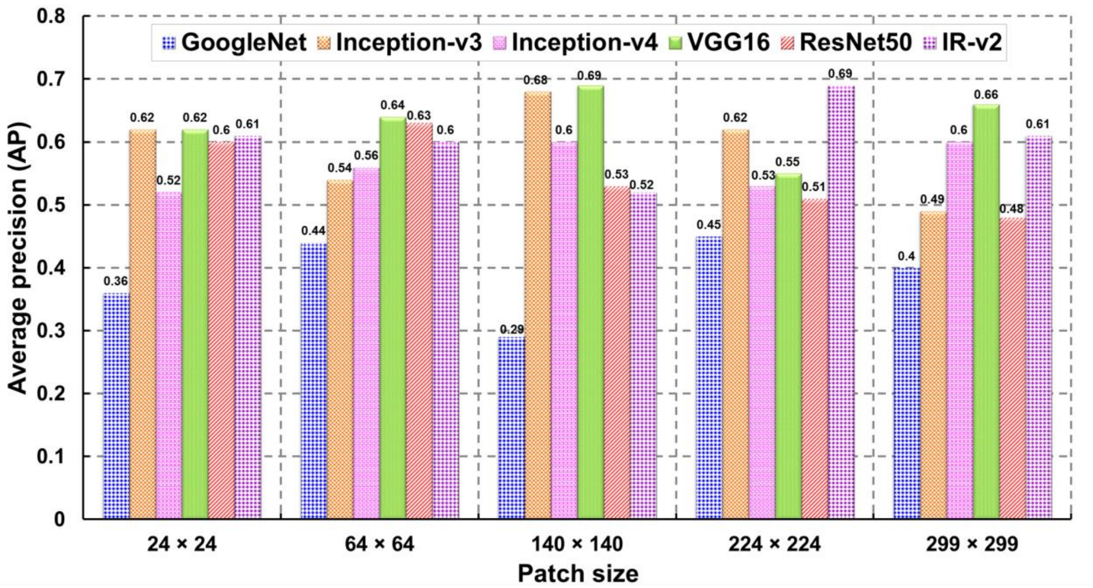

Average precision vs. patch size comparison of different pretrained models.

Supplementary Fig. S7

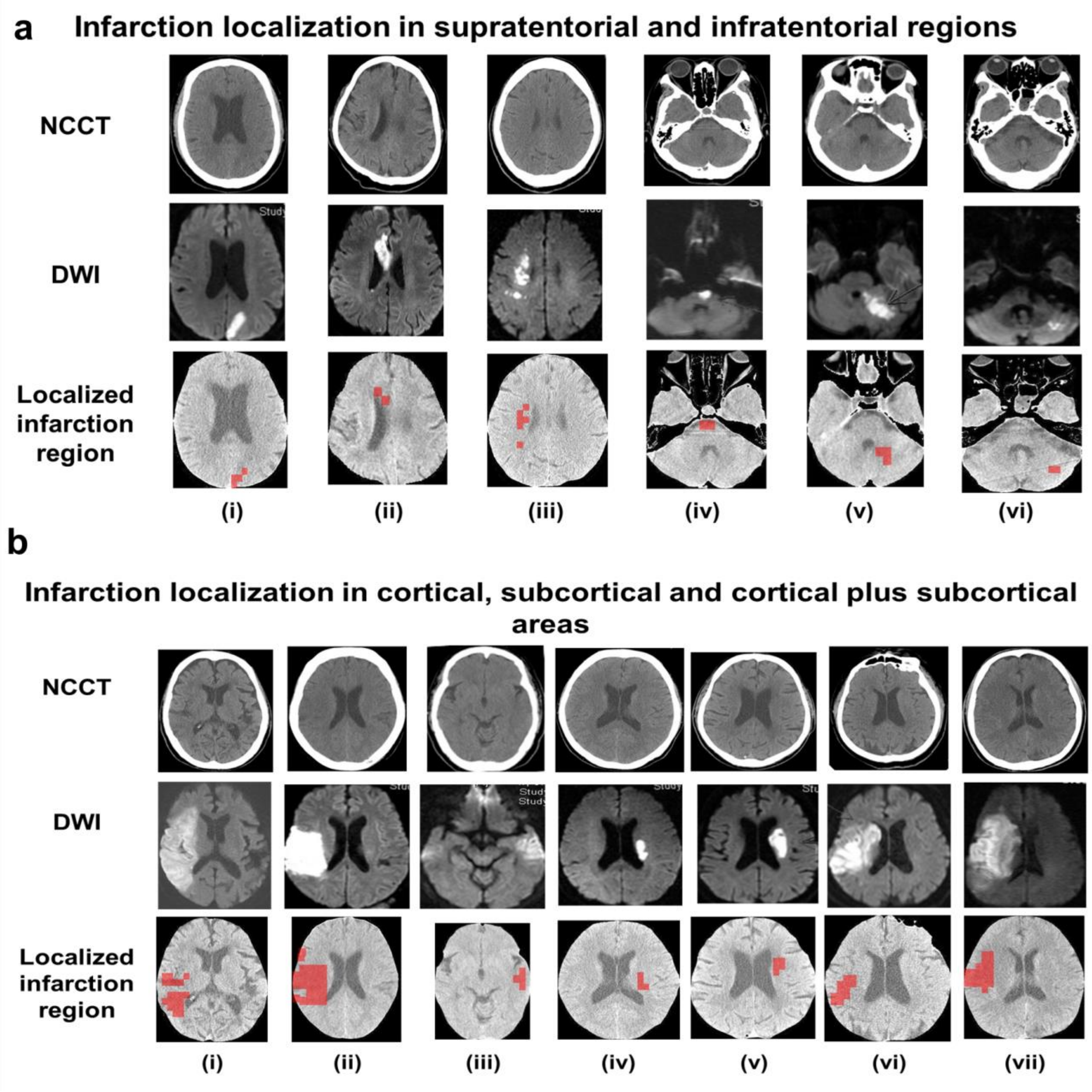

Examples of automatic infarction localization in different regions. (a) Infarction localization in supratentorial and infratentorial regions. **(b)** Infarction localization in cortical, subcortical and cortical plus subcortical areas.

NCCT, non-contrast computed tomogram; DWI, diffusion-weighted imaging.

References

1. Simonyan, K., & Zisserman, A. Very deep convolutional networks for large-scale image recognition. Preprint at https://arxiv.org/abs/1409.1556 (2014).
